# Exploring expectations of music interventions in pulmonary rehabilitation: a cross-sectional survey protocol

**DOI:** 10.1136/bmjresp-2026-004296

**Published:** 2026-08-06

**Authors:** Michel Roger Koopmans, Meike Shedden Mora, Jan Van Busschbach, Markus Klimek

**Affiliations:** 1 Department of Neuroscience, Erasmus MC, Rotterdam, The Netherlands; 2 Department Psychology, Medical School Hamburg, Hamburg, Germany; 3 Department of Psychology, Erasmus MC, Rotterdam, The Netherlands; 4 Department of Anesthesiology, Erasmus MC, Rotterdam, The Netherlands

**Keywords:** Pulmonary Rehabilitation, Pulmonary Disease, Chronic Obstructive, Psychology

## Abstract

**Introduction:**

Music interventions by listening to recorded music have been associated with the alleviation of breathlessness and the improvement of physical outcomes in the context of pulmonary rehabilitation. Results of these studies, however, should be interpreted carefully since study protocols have been heterogeneous and findings inconsistent. Studies are subject to selection bias and the effect of music interventions is assumed to depend partly on a placebo effect, increasing effectiveness when pre-treatment expectations are higher. In combination with low costs and negligible side effects, implementation of music interventions would be feasible, but they have not been structurally implemented in pulmonary rehabilitation settings yet. Before making efforts on implementing an appropriate music intervention, current use, demands and treatment expectations on music interventions of the target population and their healthcare professionals are to be explored. This study aims to explore the current use of music interventions among patients enrolled in pulmonary rehabilitation and gain insight into the expectations of patients and their caregivers regarding its effect on breathlessness and exercise tolerance.

**Methods and analysis:**

This paper describes the protocol for a cross-sectional survey consisting of a single questionnaire among patients and their healthcare professionals. The questionnaire into expectations regarding music interventions was developed and validated through a systematic and iterative process. After data collection, descriptive statistics will be performed. Cronbach’s alpha will be calculated to measure the questionnaire's internal consistency. Correlation analysis will be performed between baseline characteristics and level of expectation for music interventions.

**Ethics and dissemination:**

This study received approval of the institutional review board of the Erasmus Medical Center, The Netherlands (reference number MEC-2025-0703). Results will be disseminated through a peer-reviewed scientific publication, and raw data will be made available on reasonable request to support implementation.

**WHAT IS ALREADY KNOWN ON THIS TOPIC**

- Music interventions have been shown to alleviate breathlessness and enhance exercise tolerance. However, the applicability of previous study findings to real-world populations remains uncertain due to inherent methodological limitations. Consequently, music interventions are not yet routinely integrated into pulmonary rehabilitation practice.

**WHAT THIS STUDY ADDS**

- This study will provide insight into the expectations regarding music interventions among patients enrolled in pulmonary rehabilitation programmes as well as their healthcare providers. It will also offer an indication of the feasibility of implementing music interventions in this setting and help identify which patient groups may benefit most based on their expectation profiles.

**HOW THIS STUDY MIGHT AFFECT RESEARCH, PRACTICE OR POLICY**

- The results of this study may inform the implementation of music interventions by identifying which patients are most likely to benefit from them. In addition, the study will provide relevant insights into the preferred form and setting of music interventions from the patient perspective, thereby contributing to the design of future randomised studies.

## Introduction

### Music in pulmonary rehabilitation

 Chronic respiratory diseases account for a high disease burden, affecting more than 450 million people worldwide.^1^ Among those, chronic obstructive pulmonary disease (COPD) still contributes to the highest number of disability adjusted life years, even though its incidence is declining.^1 2^ Pulmonary rehabilitation has consistently demonstrated effectiveness in reducing hospital admissions for patients with COPD and other chronic respiratory diseases.^3 4^ It is generally recommended for individuals with either stable or unstable disease to increase fitness or patients experiencing a high symptom burden. Since pulmonary rehabilitation is characterised by its multidisciplinary approach, the scope of treatment possibilities ranges from medical and paramedical to even culturally oriented complementary therapies such as music therapy.^3 5^

Music therapy is an umbrella term encompassing many different forms of music-related interventions, either active/productive or passive/receptive. Specifically in the context of patients suffering from chronic respiratory diseases, active music interventions have shown to be effective. Among patients with COPD, multimodal musical interventions including playing wind instruments or singing have shown to increase pulmonary function outcomes, decrease shortness of breath and increase quality of life.^6–8^ Furthermore, in paediatric patients with asthma, music therapy guided by sessions with certified music therapists has shown to decrease hospital admissions and improve pulmonary function.^9^

### Music therapy versus music interventions

Despite their effectiveness, music therapy sessions, guided singing or playing wind instruments, are likely harder to structurally implement due to the need for certified musical therapists or musicians, additional time investments for patients and higher costs. Also, the interaction between the therapist and the patient can bias the outcomes. The term ‘music intervention’ is typically used to describe treatments involving only listening to recorded music.^10^ The addition of these ‘passive’/receptive music interventions to rehabilitation gained growing attention of researchers in the past few decades as well. Several systematic reviews have investigated the potential benefits of music interventions for patients with chronic respiratory diseases. Although heterogeneity among studies is considerable and findings are inconsistent, evidence suggests that music interventions with listening to recorded music could alleviate breathlessness and improve physical outcomes such as the 6 min walking tests too.^11–14^

Music interventions are generally cheap and very low on side effects, the combination of which would make implementation more feasible than active music therapy.^15^ Nevertheless, structural implementation of music interventions in guidelines in the pulmonary rehabilitation setting is still lacking.

### Challenges in music intervention research

The presence of selection bias is a widely recognised limitation of studies evaluating effects of music interventions. Patients who consent to participate in these studies are assumed to have a natural affinity for music, possibly amplifying the observed effect in the intervention arm and therefore likely limiting generalisation.^16^ Conversely, participants allocated to the control group might nevertheless still listen to music more frequently than the general population if not explicitly prohibited by the study protocol, thereby reducing between-group differences. The extent to which this selection bias influences the study outcomes is unknown and is rarely addressed in papers. Moreover, no studies have specifically quantified the impact of this selection bias as far as is known by the research team. This uncertainty on interpretation poses a significant challenge for further research or implementation.

Another important consideration when interpreting music intervention studies is the underlying mechanism responsible for the observed effect. The effect of music interventions is at least partly attributable to a placebo effect, which is known to increase with higher positive expectations regarding the intervention.^17 18^ However, results of music intervention studies are rarely corrected for treatment expectations as far as is known to the research team. This may be caused by the absence of a validated instrument to measure treatment expectations of music interventions. While several tools have been developed to assess treatment expectations in different fields of medicine, none have been specifically tailored to music-based interventions.^19–21^

Music interventions may only be beneficial for specific patient subgroups. Yet, the patient characteristics that predict favourable outcomes remain undefined. Key determinants could include not only symptom burden or expectations regarding the effect of music interventions but also cultural capital or music listening style. The term cultural capital refers to the non-financial social assets—like knowledge, skills, education and cultural tastes.^22 23^ When considering music listening style, two listening styles are distinguished in current literature: music empathising and music systemising.^24 25^ The distinction is used to address individual differences in musical experiences, preferences and other aspects of musical engagement with a specific emphasis on personality.

Before implementation can be considered, feasibility of music interventions should be assessed. Alhothaly *et al* recently published a study in which relevant factors such as current types of listening activities during exercise and genre preferences among patients in pulmonary rehabilitation programmes in the UK were studied.^26^ Importantly, they found that more than half of participants had no prior experience exercising to music. However, Alhothaly *et al* did not study patient expectations towards music interventions or perspectives of their healthcare professionals, which can be equally relevant. The belief in treatments among healthcare professionals is important to make music interventions effective and integrate them in clinical practice. A preliminary exploration by our research team found substantial variability among healthcare professionals in their views on the potential benefits of music interventions in rehabilitation setting. Addressing these knowledge gaps is essential to develop evidence-based strategies for integrating music interventions into clinical practice.

### Primary objective

The aim of this study is to explore the feasibility of implementation of music listening interventions in the setting of pulmonary rehabilitation by clarifying current use, expectations and demands of such interventions among patients and their healthcare professionals.

### Secondary objectives

- To identify associations between baseline characteristics of patients and their expectations of the effect of music interventions on breathlessness or exercise tolerance.
- To identify differences in expectations regarding music interventions among patients and their healthcare workers.

## Methods and analysis

The study protocol presented here is a cross-sectional survey among patients admitted to pulmonary rehabilitation programmes in the Netherlands and their healthcare workers. Both groups will complete a single comprehensive questionnaire designed to examine the topics of interest integrating previously validated instruments where applicable.

### Setting and participants

In the Netherlands, pulmonary rehabilitation is provided either by general hospitals, university hospitals or by specialised rehabilitation clinics. Outpatient secondary care programmes, typically provided by general hospitals, are most common. These programmes require patients to travel to the hospital or clinics several times a week to participate in exercise sessions under the supervision of specialised exercise therapists or physiotherapists. Additionally, patients attend consultations with other members of the multidisciplinary team such as psychologists or social workers. In the meantime, exercises for home training are usually provided. However, when the patient is in such condition that outpatient rehabilitation is not preferable or possible, tertiary care pulmonary rehabilitation will be offered. Provided only by the dedicated rehabilitation clinics, patients are admitted for multiple weeks (usually 8–12 weeks) to complete the rehabilitation programme. On completion of a programme, patients are referred to dedicated physiotherapists to maintain physical fitness as long as possible.

The study population comprises both patients and healthcare professionals involved in pulmonary rehabilitation. Patient inclusion criteria are as follows: (1) enrolment in a pulmonary rehabilitation programme, either inpatient or outpatient, at secondary or tertiary care level and (2) sufficient understanding of the Dutch language. Exclusion criteria for the study are (1) insufficient understanding of the Dutch language, (2) significant hearing impairment and (3) people who refuse to listen to music due to religious objections.

Healthcare professionals are eligible if they hold a medical or paramedical position at one of the study sites and are actively engaged in pulmonary rehabilitation programmes. Relevant roles include physicians, nurses, physiotherapists, exercise therapists, occupational therapists, speech therapists, psychologists, social workers and dietitians. No exclusion criteria apply for healthcare professionals.

### Study parameters and tools

We used different versions of the same questionnaire in this study: one tailored for patients and another for healthcare personnel. The patient questionnaire comprises five sections addressing the topics of (1) baseline characteristics, (2) cultural capital, (3) dyspnoea severity, (4) music listening habits, exercise and physical activity and (5) expectations from music interventions regarding dyspnoea and exercise tolerance. The version for healthcare professionals will closely mirror the patient questionnaire but will exclude sections on dyspnoea severity, music listening and physical activity. Both versions will be made available in paper format and as an online survey to maximise accessibility.

The first section collects baseline characteristics, including general demographic information such as age, gender and educational level. Depending on the version, additional items address either information on medication use or professional role within the rehabilitation programme. All gathered data will be self-reported, and no information will be extracted from medical records.

The second section covers the topic of cultural capital and music listening type. Cultural capital, a concept widely applied in social sciences, refers to the non-financial social assets—like knowledge, skills, education and cultural tastes.^22 23^ Cultural capital could be important for understanding patients’ expectations of music interventions. We want to figure out whether higher cultural capital leads to higher expectations due to more exposure and possibly more positive experiences accordingly. However, validated tools measuring cultural capital consist of long questionnaires which were considered not feasible to implement in full into our survey increasing the survey burden too much.^27^ Therefore, cultural capital will be assessed using two concise statements to which respondents will state their level of agreement on a 1–7 point Likert scale.

The construct of music listening style is commonly described in the literature through two categories: music empathisers and music systemisers.^24^ The original validated instrument to differentiate between the two comprises 18 questions to which the level of agreement is stated.^24 25^ However, similar to the approach taken for cultural capital, full implementation of this tool was deemed impractical due to concerns about survey length and respondent burden. Therefore, only the two items with the highest factor loadings for each category were selected for inclusion. Consequently, for both the measurement of cultural capital and for music listening type, the outcomes will provide an indicative trend rather than a precise measurement of these constructs.

In the third section, dyspnoea severity was assessed using the Modified Borg Dyspnea Scale, a well-validated tool often used in pulmonary medicine and palliative care to assess dyspnoea severity.^28 29^ The scale consists of two 12-point scale ratings that measure a person’s perceived breathlessness and fatigue. Additionally, four propositions are presented to which patients need to state their level of agreement on a 4-point scale. These statements cover the theme of self-influence on their feeling of breathlessness. Following the questions on dyspnoea, the fourth section covered music listening behaviours and engagement in physical activities. Outcomes will be reported using 5-point scales. Response anchors were developed in collaboration with the experts of experience to make them appropriate for the target population.

The final section of the questionnaire addresses the construct of participants’ expectations regarding the effect of music interventions. As no previously validated instruments on this topic and in this setting were known by the authors, this part of the questionnaire was separately developed specifically for the present study. The subsequent paragraphs describe the process of development of this part of the survey.

### Development and validation of the questionnaire

The final section of the survey covers several constructs regarding participants’ expectations into the effect of music interventions on breathlessness or exercise tolerance. It also addresses the perceived mechanism of effect and possible side effects. As mentioned previously, this part of the questionnaire was separately developed by the research team as no validated instruments were available for this specific context. In order to increase validity and reliability of the survey, a structured validation process was used following a method described in medical educational literature.^30^ The TEX-Q questionnaire, previously validated and translated multiple times, was used as a guideline in formulating the questions on treatment expectations.^21^ After establishing the constructs of interest, draft items were formulated which were reviewed to ensure compliance to level B1 of the Dutch language proficiency.

Following development of the preliminary version, content validity of the questionnaire was reviewed with three different experts: first an expert in the field of music intervention research was consulted (MK) followed by an independent professor in the field of medical psychology (JVB). Finally, an expert in the field of treatment expectations and developer of the TEX-Q questionnaire was consulted (MSM). In personal meetings with the research team, the topics, constructs, questions, clarity and response anchors were discussed subsequently revising and optimising the questionnaire.

After initial expert consultation, the questionnaire was evaluated with experts by experience. By collaborating with the largest patient organisation for respiratory diseases, the Lung Foundation Netherlands (Longfonds), patients who had previously participated in pulmonary rehabilitation programmes were consulted. In individual sessions with a member of the research team, the questionnaire was reviewed and following a structured format, the content, comprehensibility of the questions and its response anchors were discussed. All feedback was documented and reviewed by the research team. Where relevant and applicable, questions were altered, added or removed hence resulting in an updated version of the questionnaire.

The updated version of the questionnaire underwent a final review with two experts (MK and MSM) after which it was presented and tested once more with five different experts by experience. The latter were this time recruited from the ward of pulmonary medicine of the Erasmus Medical Center. Unlike the first round, which primarily involved volunteers with an affinity with music, the second group of experts by experience was a more heterogeneous sample of patients making it more representative for the study setting. Following this round of review, no significant alterations were made to the questionnaire anymore. This self-developed section has not yet been formally tested in a pilot sample in order to determine internal reliability. It will be used in a larger patient population for the first time in the context of this study and thus, a Cronbach’s alpha coefficient has not yet been calculated.^31^ After data collection, a Cronbach’s alpha coefficient will be calculated to finalise the validation process.

The final questionnaire on music intervention-related expectations consists of 27 propositions and two multiple choice questions. The topics covered are fourfold: (1) expectations of music interventions on breathlessness, (2) expectations of music interventions on exercise tolerance, (3) side effects of music interventions and (4) expected mechanism of action of music interventions in the theme of breathlessness or exercise tolerance. Level of agreement is measured using a 1 (fully disagree) to 4 (fully agree) Likert scale. In order to increase reliability, the propositions are phrased in the first person (eg, ‘…for my breathlessness’). This phrasing would, however, not always be appropriate for the healthcare professionals. Therefore, when applicable, the questions for the healthcare professionals are stated in the third person, referring to their patient instead of oneself, making the exact statements slightly different.

The Dutch questionnaires are available in the online supplemental materials. However, in order to increase scientific transparency, we have added an English version of the questionnaire as well in order to enable non-Dutch speaking readers to gain insight into the researched constructs in this study. Translation of the questionnaire was performed using forward and backward translation. The latter of which was performed by an independent researcher that was not previously involved in the development of the questionnaire. It should be noted that the English version of the questionnaire has not been subject to testing or validation in pilot samples, and no psychometric data on this version are obtained. It should be used solely as a tool to increase clarity for all readers of this paper.

### Patient and public involvement

Patient involvement played a central role in the development of this study. Patients, experts by experience in pulmonary rehabilitation, were engaged at multiple stages of the questionnaire validation process. In the initial development stage, a call for voluntary participation was disseminated through the official communication channels of the Lung Foundation Netherlands (Longfonds). All patients who responded were invited to participate in individual online meetings with the researcher.

During these meetings, the questionnaire was evaluated with regard to its content and perceived relevance. Patient experiences were incorporated, and discussions extended beyond question content to include vocabulary, phrasing and response anchors. In this phase, five patients contributed actively to shaping the questionnaire.

In a later phase, additional patients were recruited from the pulmonary medicine ward at the Erasmus Medical Center, Rotterdam, to voluntarily contribute to the further development of the questionnaire. Their involvement in this stage supported the refinement of question clarity and the optimisation of the questionnaire’s formatting. Although no substantial changes to the overall content were made at this point, their feedback was instrumental in enhancing the usability and accessibility of the questionnaire for a broader patient population.

### Data collection and recruitment

Data collection will be conducted over a period of two predetermined weeks at each study site. During this period, members of the research team will visit the different study sites and approach potential participants in person, inviting them for participation. Recruitment will primarily occur after group exercise sessions or during ward visits for patients admitted to rehabilitation clinics. Specific recruitment times will be coordinated in close collaboration with each site. Patients who consent to participate may choose to complete the questionnaire either online or in paper format.

Healthcare personnel will be recruited primarily via email. Email lists will be compiled in cooperation with the participating institutions to identify all staff involved in the pulmonary rehabilitation programmes. Where possible, healthcare workers too will be recruited in person and will be provided with the choice to either fill out the questionnaire online or on paper. In both groups, the online surveys will be available using Microsoft forms, and the data capture programme used is Castor.

### Sample size

The primary objective of this study is to obtain a representative sample of patients currently enrolled in pulmonary rehabilitation programmes. Since no intervention is being tested, a power analysis is not performed. However, it is aimed to reach a target response rate of 40% of all patients admitted to the pulmonary rehabilitation programmes during the weeks of data collection. For healthcare professionals, the target response rate is set at 60%.

### Analysis

R V.3.6.3 (R Foundation for Statistical Computing, Vienna, Austria) or higher will be used for data analysis. The significance level of all tests will be set at <0.05. For the majority of data, descriptive statistics will be used, but correlation tests will also be performed for the secondary objectives of this study.

When the level of agreement to a proposition is measured, 1–4 Likert scale results will be treated as ordinal variables. When frequencies are measured, they will be treated as categorical variables. The data will be assessed for normal distribution. Depending on the type of distribution found, random or fixed models will be used to investigate correlation between answers.

Normally distributed data will be analysed using the Student’s t-test. Data will be represented as means with their SD (±SD). Not normally distributed continuous data will be analysed using the non-parametric Mann-Whitney U test. Outcomes will be presented as medians and their IQRs. Normality of continuous variables will be assessed with the Shapiro-Wilk test and graphically in histograms. Homogeneity of variances will be tested using the Levene’s test. Categorical data will be analysed using the χ^2^ test.

Correlation tests will be performed to analyse associations between baseline characteristics, cultural capital, music listening type, dyspnoea severity and specific expectations-propositions. Depending on the type of variable (continuous or categorical), either t-tests, logistic or linear regression analysis, respectively will be used.

### Burden and risk

No significant burden is associated with this research. The questionnaire will take 10–15 min for patients to complete and 5–10 min for healthcare workers. Usual care will not be altered when participating in this study.

### Potential limitations, strengths and future use

This study has a few potential limitations to take into account. The most important limitation of the study is the fact that for the main construct of interest, a new questionnaire had to be developed which was not previously validated. Consequently, certain reliability parameters remain unknown until completion of data collection, as the instrument will be tested in the context of this study for the first time. Internal consistency will be assessed post hoc using Cronbach’s alpha. Nevertheless, efforts have been made to maximise content validity of the questionnaire by putting it through several rounds of review with professional experts as well as with experts by experience. Therefore, the research team believes the questionnaire to sufficiently assess the different constructs of interest.

Another limitation concerns the measurements of the operationalised factors such as cultural capital and music listening style. For feasibility reasons, not the complete validated instruments are implemented in the survey. Instead, only those parts that are considered most relevant to our survey are used resulting in the fact that outcomes of these parameters should be merely interpreted as indicative, and not as a valid representative measurement of the intended constructs. Correlation analysis shall therefore be interpreted with care when performed with these constructs.

A final limitation of this study is its potential selection bias. Although the study will not be presented as a study into ‘music interventions’, but a study into expectations of ‘non-pharmacological interventions’, a risk of selection bias will nevertheless exist with this framing method. By recruiting patients in person, the research team aims to mitigate this risk of bias since the framing will be done in a standardised way by the research team itself ensuring consistent communication across sites.

In contrast, this study also knows several strengths. To our knowledge, it is the first investigation into expectations of music interventions among patients undergoing pulmonary rehabilitation. The findings will provide valuable information regarding the feasibility of implementation and the goals for which future music interventions could best be designed. It might provide insight into patient subgroups with the highest expectations from music interventions, who consequently could possibly benefit most due to an increased placebo effect.

In the introduction of this paper, two methodological challenges in music intervention studies are introduced: the selection bias and the placebo effect. In the future, the questionnaire developed for this study could be valuable in addressing both of these problems. If the questionnaire after this validation study finally will be employed as a baseline measurement for future music intervention studies, it will enable comparisons between included patients and the general population of pulmonary rehabilitation patients, providing insight into the selection bias that might have occurred during the recruitment of patients. Possibly, expectations on music interventions of participants included in new randomised trials are higher than those of the general population, thus confounding outcomes. By incorporating this questionnaire as a baseline assessment, researchers can quantify the extent of selection bias.

Furthermore, because patient expectations will be measured on an ordinal scale in this survey, future analysis could be corrected for patients’ baseline expectations. This might provide valuable information on how treatment expectations influence outcomes, possible discriminating between placebo and an inherent treatment effect. Thus, not only the selection bias, but also the possible placebo effect could be reviewed in a quantitative way when this questionnaire is used in future research. Since selection bias and placebo by expectation are common limitations of many (music intervention-) studies, we advocate measurements of treatment expectations before every music intervention study using the questionnaire developed for this study after completion of its validation process.

## Ethics and dissemination

This study received a non-WMO (Wet Medisch-wetenschappelijk Onderzoek met mensen) declaration of the Medical ethical committee of the Erasmus Medical Center, Rotterdam, The Netherlands, confirming that it does not fall under the Dutch Medical Research involving Human Subjects Act (WMO). Furthermore, an exemption from written informed consent was requested and granted by the same IRB. The study will apply to rules of the General Data Protection Regulation. Survey data will be collected fully anonymously. No personal information will be gathered in the process of this research and once respondents complete the questionnaire and provide it to the research team, their answers will not be traceable anymore.

Study results will be published in a peer-reviewed scientific paper. On request, raw data will be available for the development of new studies or implementation purposes on contact with the research team.

## Supplementary material

10.1136/bmjresp-2026-004296 online supplemental file 1

## Data Availability

Data are available upon reasonable request.
