# Supplementary material for: Exploring expectations of music interventions in pulmonary rehabilitation: a cross-sectional survey protocol
Source: BMJ Open Respir Res. 2026 Aug 6;13(1):e004296. doi: 10.1136/bmjresp-2026-004296 (PMC13448574; doi:10.1136/bmjresp-2026-004296)
Supplement: Supplementary file 1 [file bmjresp-13-1-s001.pdf]

-Supplementary material 1-

Title: Questionnaire for patients. Dutch version.

Note: The English versions of the questionnaires are included later in this document.

Onderdeel 1: Algemene vragen

Hieronder staan een aantal algemene vragen over uw situatie. Beantwoord de vragen.

Wat is uw leeftijd? ______

Wat is uw geslacht?

- Man
- Vrouw
- Wil ik liever niet zeggen
- Anders namelijk: ____

Waarom revalideert u op dit moment? Geef de belangrijkste reden.

- COPD
- Astma
- Longfibrose
- Pulmonale hypertensie
- Longkanker
- (herhaalde) Longontsteking
- Bronchiectasieën
- Longtransplantatie
- Anders namelijk: ____

Wat is uw hoogst behaalde opleidingsniveau?

- Basisonderwijs
- Lager beroepsonderwijs of vergelijkbaar (zoals Lts, huishoudonderwijs, lager land- en tuinbouwonderwijs)
- Mavo, vmbo of vergelijkbaar
- Havo vwo of vergelijkbaar
- Middelbaar beroepsonderwijs of vergelijkbaar (zoals mbo, meao, middelbaar land- en tuinbouwonderwijs_
- Hoger beroepsonderwijs of vergelijkbaar (zoals hbo, hts, heao)
- Universiteit
- Anders namelijk: ____

Revalideert u klinisch of poliklinisch?

- Klinisch. Ik slaap in het revalidatiecentrum.
- Poliklinisch. Ik slaap thuis, maar kom voor de behandelingen naar het revalidatiecentrum.

Hoeveel ver bent u al in het revalidatie traject? Vul de onderstaande zin aan.

Ik zit in week _____ van totaal ____ weken revalidatie.

Gebruikt u dagelijks inhalatiemedicatie (puffers of vernevelingen)?

- Ja
- Nee

Hoeveel dosis inhalatiemedicatie gebruikt u <u>vast</u> per dag? Tel het totale aantal dosis bij elkaar op.	

Hoeveel dosis inhalatiemedicatie gebruikt u <u>zo nodig extra</u> (rescue-medicatie) per dag? Tel het totale aantal extra dosis bij elkaar op.	

Hoeveel <u>verschillende</u> <u>soorten</u> medicijnen gebruikt u in totaal? (Niet alleen voor uw longziekte, maar voor alle mogelijke aandoeningen!)

- 0
- 1-2
- 2-4
- 5-6
- 6-8
- >8

Geef aan in hoeverre u het oneens of eens bent met onderstaande stellingen.

	Helemaal mee oneens	Mee oneens	Enigszins oneens	Neutraal	Enigszins eens	Mee eens	Helemaal mee eens
Ik zie mijzelf als een liefhebber van kunst en cultuur.	○	○	○	○	○	○	○
Ik zie mijzelf als een liefhebber van muziek.	○	○	○	○	○	○	○

Bent u professioneel musicus?	○ Ja	○ Nee

	Nooit	Enkele keer per maand	Meermaals per week	Dagelijks
Hoe vaak bespeelt u een instrument?	○	○	○	○
Hoe vaak zingt u?	○	○	○	○

Hieronder staan een aantal vragen die gaan over uw muziek luisterstijl. Geef aan in hoeverre u het oneens of eens bent met onderstaande stellingen.

	Helemaal mee oneens	Oneens	Eens	Helemaal mee eens
Ik denk dat ik, wanneer ik naar muziek luister, de emoties kan begrijpen die de schrijver of uitvoerder probeert uit te drukken.	○	○	○	○
Tijdens het luisteren naar muziek vraag ik me af hoe de schrijver of componist zich toen voelde.	○	○	○	○
Ik waardeer het om de verschillende lagen van instrumenten en zang in een muziekstuk te horen.	○	○	○	○
Ik vraag me vaak af hoe de mechaniek van muziekinstrumenten werkt.	○	○	○	○

Onderdeel 2: Kortademigheid.

Hieronder staan een aantal vragen over uw kortademigheid. Beantwoord eerst de vragen en geef daarna aan in hoeverre u het oneens of eens bent met onderstaande stellingen.

Geef je gemiddelde kortademigheid/dyspnoe over de dag een cijfer (omcirkel).

0	0.5	1	2	3	4	5	6	7	8	9	10
Helemaal niet	Nagenoeg niet	Zeer weinig	Weinig	Matig	Tamelijk sterk	Sterk		Zeer sterk			Helemaal geen adem

Geef je gemiddelde vermoeidheid/zwaarte over de dag een cijfer (omcirkel).

0	0.5	1	2	3	4	5	6	7	8	9	10
Niet voelbaar	Heel erg licht	Erg licht	Licht	Matig	Redelijk zwaar	Zwaar		Erg zwaar			Heel erg zwaar, maximaal

	Helemaal mee oneens	Oneens	Eens	Helemaal mee eens
Ik heb zelf invloed heb op hoe kortademig ik mij voel.	○	○	○	○
Ik ken genoeg manieren om mijzelf minder kortademig te voelen.	○	○	○	○
Ik sta open voor nieuwe manieren om me minder kortademig te voelen.	○	○	○	○
Ik ben bereid om dingen anders te doen als dat helpt tegen mijn kortademigheid.	○	○	○	○

Welke dingen, anders dan medicijnen, gebruikt u om uzelf minder kortademig te voelen?

(schrijf zo veel mogelijk dingen op

Onderdeel 3: Muziek als behandeling.

Mogelijk kunnen behandelingen met muziek, zoals muziek luisteren of maken, invloed hebben op het gevoel van kortademigheid. Hieronder staan een aantal stellingen over uw verwachtingen van het effect van muziek op kortademigheid. Geef aan in hoeverre u het oneens of eens bent met de stellingen.

	**Helemaal mee oneens**	**Oneens**	**Eens**	**Helemaal mee eens**
Ik denk dat muziek luisteren mijn gevoel van kortademigheid kan verminderen.	○	○	○	○
Ik denk dat zingen mijn gevoel van kortademigheid kan verminderen.	○	○	○	○
Ik denk dat het maken van muziek mijn gevoel van kortademigheid kan verminderen.	○	○	○	○
Ik kan mijn kortademigheid beter verdragen als ik muziek luister.	○	○	○	○
Ik denk dat het van meerwaarde is om muziek te luisteren wanneer ik mij kortademig voel.	○	○	○	○
Ik zou mensen in mijn situatie aanraden om muziek te luisteren als zij zich kortademig voelen.	○	○	○	○

Hieronder staan een aantal stellingen over welk effect het luisteren van muziek op u kan hebben. Geef aan in hoeverre u het oneens of eens bent met de stellingen.

	**Helemaal mee oneens**	**Oneens**	**Eens**	**Helemaal mee eens**
Muziek luisteren zorgt voor afleiding van mijn gevoel van kortademigheid.	○	○	○	○
Muziek luisteren ontspant mij.	○	○	○	○
Wanneer ik naar muziek luister, ervaar ik doorgaans meer positieve gevoelens.	○	○	○	○
Wanneer ik muziek luister, kan ik mij beter concentreren op mijn taak op dat moment.	○	○	○	○
Als ik naar muziek luister, ga ik doorgaans mee ademen met het ritme van de muziek.	○	○	○	○

Hoeveel voordeel verwacht u van het actief luisteren van muziek tegen het gevoel van kortademigheid?

0. Geen voordeel					5. Neutraal					10. Maximaal voordeel
○	○	○	○	○	○	○	○	○	○	○

Vraag: Stel, u voelt zich kortademig. U wilt proberen of muziek luisteren helpt. Welke muziek zou u dan kiezen? Kies het best passende antwoord.

- ○ Ritmische, up-beat muziek
- ○ Niet-ritmische muziek, bijvoorbeeld meditatie- of natuurgeluiden
- ○ Mijn eigen favoriete muziek
- ○ De muziek die mijn behandelaar mij daarvoor adviseert
- ○ Anders namelijk: ____

____

Mogelijk kan het luisteren van muziek invloed hebben op hoe goed u kunt bewegen. Dat geldt bijvoorbeeld op het moment dat u sportoefeningen doet, wandelt, traploopt of fysieke taken moet doen zoals zelfzorg of het huishouden. Onderstaande vragen gaan over het effect van muziek op het bewegen.
Geef aan in hoeverre u het oneens of eens bent met onderstaande stellingen.

	Helemaal mee oneens	Oneens	Eens	Helemaal mee eens
Ik denk dat het luisteren van muziek mij helpt om beter te bewegen.	○	○	○	○
Ik denk dat ik beter kan presteren als ik muziek luister tijdens het bewegen.	○	○	○	○
Ik denk dat ik mij overvraag als ik muziek luister tijdens het bewegen. Ik beweeg dan te veel en dat werkt mij tegen.	○	○	○	○
Ik zou mensen in mijn situatie aanraden om muziek te luisteren tijdens het bewegen.	○	○	○	○
Als ik muziek luister tijdens het inspannen, ga ik mee bewegen met het ritme van de muziek.	○	○	○	○

Hoe veel voordeel verwacht u van het luisteren van muziek tijdens het bewegen?

0. Geen voordeel — 5. Neutraal — 10. Maximaal voordeel

○ ○ ○ ○ ○ ○ ○ ○ ○ ○ ○

Stel: u bent bij de fysiotherapeut. U wilt proberen of muziek luisteren u helpt om beter te kunnen bewegen. Welke muziek zou u dan kiezen? Kies alleen 1 antwoord!

- ○ Ritmische, up-beat muziek
- ○ Niet-ritmische muziek, bijvoorbeeld meditatie- of natuurgeluiden
- ○ Mijn eigen favoriete muziek
- ○ Muziek die mijn behandelaar mij daarvoor adviseert
- ○ Anders namelijk: ____________________

Onderdeel 4: Nadelen van muziek luisteren.

Muziek luisteren kent bijna geen risico's. Toch zijn er redenen waarom mensen het luisteren van muziek niet altijd prettig vinden. De volgende vragen gaan over eventuele ongewenste effecten van het luisteren van muziek.

	Helemaal mee oneens	Oneens	Eens	Helemaal mee eens
Muziek luisteren met een koptelefoon verhindert het contact met de mensen om mij heen.	○	○	○	○
Wanneer er muziek op staat in de ruimte, verhindert dat het contact met de mensen om mij heen.	○	○	○	○
Muziek luisteren maakt mij minder bewust van mijn omgeving.	○	○	○	○
Muziek luisteren tijdens het doen van een taak zorgt er doorgaans voor dat ik mij minder goed kan concentreren.	○	○	○	○
Als ik muziek luister terwijl ik me niet goed voel, kan ik die muziek gaan verbinden met negatieve gevoelens.	○	○	○	○
Ik vind het vervelend om oordopjes voor muziek in te hebben.	○	○	○	○
Ik vind het vervelend om een koptelefoon op te hebben.	○	○	○	○
Ik kan mij storen aan muziek als deze op staat in de ruimte.	○	○	○	○

In welke mate verwacht u dat het actief luisteren van muziek als behandeling u zal beperken bij uw andere normale bezigheden in het dagelijks leven?

0. Helemaal niet — 5. Neutraal — 10. extreem veel

○	○	○	○	○	○	○	○	○	○	○

Onderdeel 5: muziek en sport.

Hieronder staan een aantal vragen over hoe veel tijd u aan bewegen en muziek besteedt. Beantwoord de vragen.

**Hoeveel tijd per week besteed u gemiddeld aan…**	**Dit doe ik zelden tot nooit**	**Maximaal 1 uur**	**Maximaal 3 uur**	**Maximaal 5 uur**	**Meer dan 5 uur**
…sportoefeningen binnenshuis, zelfstandig?	○	○	○	○	○
…sportoefeningen buiten, zoals wandelen of fietsen, zonder begeleiding van een fysiotherapeut/trainer?	○	○	○	○	○
…sportoefeningen in groepsverband of met de fysiotherapeut/trainer?	○	○	○	○	○
**Hoeveel tijd per dag gemiddeld luistert u muziek….**					
…terwijl u iets anders doet (bijvoorbeeld schoonmaken, werken, autorijden etc)?	○	○	○	○	○
…terwijl u niets anders doet (u gaat bewust zitten om de muziek te luisteren)?	○	○	○	○	○
**Staat er muziek aan, of zet u muziek op wanneer u…**	**Zelden tot nooit**	**Minder dan de helft van de tijd**	**Ongeveer de helft van de tijd**	**Meer dan de helft van de tijd**	**Bijna altijd tot altijd**
…sportoefeningen doet binnenshuis, zelfstandig?	○	○	○	○	○
…sportoefeningen doet buitenshuis zelfstandig zoals wandelen of fietsen, zonder begeleiding van een fysiotherapeut/trainer?	○	○	○	○	○
…sportoefeningen doet in groepsverband of met fysiotherapeut/trainer?	○	○	○	○	○

Wanneer u muziek luistert, via welk platform luistert u dan meestal naar muziek?

- ○ Ik luister geen muziek.
- ○ Ik gebruik meestal een CD speler of Platenspeler.
- ○ Ik luister meestal muziek op online audio streamingsdiensten.
- ○ Ik luister meestal muziek via online video streamingsdiensten.
- ○ Ik luister meestal muziek op de radio.
- ○ Ik luister meestal live muziek.
- ○ Anders, namelijk: ____

Wanneer u muziek luistert, hoe luistert u dan het liefst muziek?

- ○ Ik luister nooit muziek.
- ○ Ik zet de muziek het liefst hardop aan op de boxen in de ruimte.
- ○ Ik gebruik het liefst oortjes.
- ○ Ik gebruik het liefst een koptelefoon.
- ○ Anders, namelijk: ____

De vragenlijst eindigt met een aantal algemene vragen. Beantwoord de vragen.

	Ja	Nee
Heeft u ooit een vorm van muziektherapie aangeboden gekregen bij de behandeling van uw ziekte?	○	○
Als uw antwoord op de vorige vraag 'ja' was: heeft u daar gebruik van gemaakt?	○	○
Heeft u het luisteren van muziek ooit als behandeling tegen uw kortademigheid gebruikt?	○	○
Heeft u zingen ooit als behandeling tegen uw kortademigheid gebruikt?	○	○
Zou u bereid zijn om mee te doen aan een behandeling met muziektherapie als u dit zou worden aangeboden.	○	○
Zou u bereid zijn mee te doen aan een studie om te onderzoeken wat het effect is van muziek is binnen de longrevalidatie. (*Deze vraag is alleen om te kijken of u interesse heeft. Als u 'ja' zegt, verplicht u zich nog nergens toe. Wij kunnen niet zien, wie deze antwoord geeft en zullen u ook niet naar aanleiding van uw antwoord benaderen!)*	○	○
Heeft deze vragenlijst u geprikkeld om zelf muziek te gaan proberen tegen het gevoel van kortademigheid of om beter te kunnen bewegen.	○	○

Heeft u nog overige opmerkingen die u met de onderzoeker zou willen delen? Schrijft deze dan hier op.

Supplementary material 2

Title: Questionnaire for health care professionals. Dutch version.

Onderdeel 1: Algemene vragen

Hieronder staan een aantal algemene vragen over uw situatie. Beantwoord de vragen.

Wat is uw leeftijd? ______

Wat is uw geslacht?

- ○ Man
- ○ Vrouw
- ○ Wil ik liever niet zeggen
- ○ Anders namelijk: ____

Wat is uw functie?

- ○ Longarts
- ○ Revalidatiearts
- ○ Arts-assistent
- ○ Fysiotherapeut
- ○ Ergotherapeut
- ○ Logopedist
- ○ Oefentherapeut
- ○ Creatief therapeut
- ○ Diëtist
- ○ Anders, namelijk: ____

	Helemaal mee oneens	Mee oneens	Enigszins oneens	Neutraal	Enigszins eens	Mee eens	Helemaal mee eens
Ik zie mijzelf als een liefhebber van kunst en cultuur.	○	○	○	○	○	○	○
Ik zie mijzelf als een liefhebber van muziek.	○	○	○	○	○	○	○

Bent u professioneel musicus?	○ Ja ○ Nee

**Hoeveel tijd per dag luistert u muziek….**	Dit doe ik zelden tot nooit	Maximaal 1 uur	Maximaal 3 uur	Maximaal 5 uur	Meer dan 5 uur
…terwijl u <u>iets anders</u> doet (bijvoorbeeld schoonmaken, werken, autorijden etc)?	○	○	○	○	○
…terwijl u <u>niets anders</u> doet (u gaat bewust zitten om de muziek te luisteren)?	○	○	○	○	○

	Nooit	Enkele keer per maand	Meerdere keer per week	Dagelijks
Hoe vaak bespeelt u een instrument?	○	○	○	○
Hoe vaak zingt u?	○	○	○	○

Hieronder staan een aantal vragen die gaan over uw muziek luisterstijl. Geef aan in hoeverre u het oneens of eens bent met onderstaande stellingen.

	Helemaal mee oneens	Oneens	Eens	Helemaal mee eens
Ik denk dat ik, wanneer ik naar muziek luister, de emoties kan begrijpen die de schrijver of uitvoerder probeert uit te drukken.	○	○	○	○
Tijdens het luisteren naar muziek vraag ik me af hoe de schrijver of componist zich toen voelde.	○	○	○	○
Ik waardeer het om de verschillende lagen van instrumenten en zang in een muziekstuk te horen.	○	○	○	○
Ik vraag me vaak af hoe de mechaniek van muziekinstrumenten werkt.	○	○	○	○

Wanneer u muziek luistert, via welk platform luistert u dan meestal naar muziek

- ○ Ik luister geen muziek.
- ○ Ik gebruik meestal een CD speler of Platenspeler.
- ○ Ik luister meestal muziek op online audio streamingsdiensten.
- ○ Ik luister meestal muziek via online video streamingsdiensten.
- ○ Ik luister meestal muziek op de radio.
- ○ Ik luister meestal live muziek.
- ○ Anders, namelijk: ____

Wanneer u muziek luistert, hoe luistert u dan het liefst muziek?

- ○ Ik luister nooit muziek.
- ○ Ik zet de muziek het liefst hardop aan op de boxen in de ruimte.
- ○ Ik gebruik het liefst oortjes.
- ○ Ik gebruik het liefst een koptelefoon.
- ○ Anders, namelijk: ____

Onderdeel 2: verwachtingen van muziek als behandeling

Geef aan in hoeverre u het oneens of eens bent met onderstaande stellingen.

	**Helemaal mee oneens**	**Oneens**	**Eens**	**Helemaal mee eens**
Ik denk dat muziek luisteren het gevoel van kortademigheid kan verminderen.	○	○	○	○
Ik denk dat zingen het gevoel van kortademigheid kan verminderen.	○	○	○	○
Ik denk dat het maken van muziek het gevoel van kortademigheid kan verminderen.	○	○	○	○
Ik denk dat het van meerwaarde is om muziek te luisteren wanneer ik mijn patiënt zich kortademig voelt.	○	○	○	○
Ik zou mensen in mijn situatie aanraden om muziek te luisteren als zij zich kortademig voelen.	○	○	○	○

	**Helemaal mee oneens**	**Oneens**	**Eens**	**Helemaal mee eens**
Muziek luisteren zorgt voor afleiding van het gevoel van kortademigheid.	○	○	○	○
Muziek luisteren ontspant.	○	○	○	○
Wanneer je naar muziek luistert, ervaar je doorgaans meer positieve gevoelens.	○	○	○	○
Wanneer mijn patiënt muziek luistert, kan die zich beter concentreren op mijn taak.	○	○	○	○
Als je naar muziek luistert, ga je mee ademen met het ritme van de muziek.	○	○	○	○

Hoeveel voordeel verwacht u van het luisteren van muziek tegen het gevoel van kortademigheid?

0. Geen voordeel					5. Neutraal					10. Maximaal voordeel
○	○	○	○	○	○	○	○	○	○	○

Vraag: Stel, u patiënt is benauwd. U wilt proberen of muziek luisteren helpt. Welke muziek zou u dan kiezen? Kruis aan.

- ○ Ritmische, up-beat muziek
- ○ Niet-ritmische muziek, bijvoorbeeld meditatiegeluiden.
- ○ Zijn eigen favoriete muziek
- ○ Muziek die de wij als behandelaars daar het meest geschikt voor vinden.
- ○ Anders namelijk: ____

Mogelijk kan het luisteren van muziek invloed hebben op hoe goed een patiënt kan bewegen. Dat geldt bijvoorbeeld op het moment dat hij/zij sportoefeningen doet, wandelt, traploopt of fysieke taken moet doen zoals zelfzorg of het huishouden.
Onderstaande vragen gaan over het effect van muziek op het bewegen.
Geef aan in hoeverre u het oneens of eens bent met onderstaande stellingen.

	Helemaal mee oneens	Oneens	Eens	Helemaal mee eens
Ik denk dat het luisteren van muziek mijn patiënt helpt om beter te bewegen.	○	○	○	○
Ik denk dat mijn patiënt beter kan presteren als ik muziek luister tijdens het bewegen.	○	○	○	○
Ik denk dat ik mijn patiënt zich overvraagt als die muziek luistert tijdens het bewegen. Hij/zij beweeg dan te veel en dat werkt hem/haar tegen.	○	○	○	○
Ik zou mijn patienten in de longrevalidatie aanraden om muziek te luisteren tijdens het sporten.	○	○	○	○
Als je muziek luistert tijdens het bewegen, ga je mee bewegen met het ritme van de muziek.	○	○	○	○

Hoe veel voordeel verwacht u van het luisteren van muziek tijdens het bewegen?

0. Geen voordeel					5. Neutraal					10. Maximaal voordeel
○	○	○	○	○	○	○	○	○	○	○

*Stel: u bent de oefentherapeut. U wilt proberen of muziek helpt om de patiënt beter te laten bewegen. Welke muziek zou u dan kiezen?

- ○ Ritmische, up-beat muziek.
- ○ Niet-ritmische muziek, bijvoorbeeld meditatiegeluiden.
- ○ Zijn eigen favoriete muziek.
- ○ Muziek die wij daar als behandelaars het meest geschikt voor vinden.
- ○ Anders namelijk: ____

Onderdeel 4: Nadelen van muziek luisteren.

Muziek luisteren kent bijna geen risico's. Toch zijn er redenen waarom mensen het luisteren van muziek niet altijd prettig vinden.
De volgende vragen gaan over eventuele ongewenste effecten van het luisteren van muziek.

	Helemaal mee oneens	Oneens	Eens	Helemaal mee eens
Muziek luisteren met een koptelefoon verhindert het contact van de patiënt met de mensen om hem/haar heen.	○	○	○	○
Wanneer er muziek op staat in de ruimte, verhindert dat het contact van de patiënt met de mensen om hem/haar heen.	○	○	○	○
Muziek luisteren maakt de patiënt minder bewust van mijn omgeving.	○	○	○	○
Muziek luisteren zorgt ervoor dat mijn patiënt zich minder goed kan concentreren.	○	○	○	○
Als mijn patiënt muziek luistert terwijl hij/zij zicht niet goed voelt, kan die die muziek gaan verbinden met negatieve gevoelens.".	○	○	○	○
Ik vind het vervelend om oordopjes voor muziek in te hebben.	○	○	○	○

Ik vind het vervelend om een koptelefoon op te hebben.	○	○	○	○
Ik kan mij irriteren aan muziek als deze op staat in de ruimte.	○	○	○	○
Ik denk dat een vorm van implementatie van muziek als behandeling meerwaarde kan hebben voor longpatiënten.	○	○	○	○

**In welke mate verwacht u dat het van luisteren van muziek als behandeling de patiënt zal beperken in het andere taken in het dagelijks leven?

0. Helemaal niet — 5. Neutraal — 10. extreem veel

○ ○ ○ ○ ○ ○ ○ ○ ○ ○ ○

Heeft deze vragenlijst u geprikkeld om zelf muziek te gaan proberen tijdens de behandeling?

- ○ Ja
- ○ Nee

COMPOSITION questionnaire

For patients.

Dear Sir/Madam,

Welcome, and thank you for participating.

Purpose of the Study
This questionnaire explores your expectations regarding non-pharmacological, complementary treatments aimed at improving breathlessness or enhancing mobility.

How Does It Work?
You will complete a single questionnaire consisting of five sections. It starts with general questions, followed by questions about your breathlessness and physical activity. Next, we ask for your opinion on non-pharmacological, complementary treatments. Please answer the questions with whatever comes to mind first. There are no right or wrong answers. Even if you haven't thought about these topics before, your opinion is still valuable. You may choose to fill out the questionnaire yourself or have a member of the research team ask you the questions. Participation takes about 10–15 minutes, and your responses will help improve care for people with lung conditions.

Risks
There are no risks associated with this study.

Data
The questionnaire is completed entirely anonymously, and your answers cannot be traced back to you. We do not have access to your medical records and will not extract any data from them. All information will be stored securely.

Important Information
Participation is voluntary and will not affect the quality of your care. Your answers will not influence the treatment or care you currently receive. However, we hope your input will help improve future care. You may stop participating at any time without giving a reason. You may also skip any question you prefer not to answer. We do not collect any personal data. Once you submit or hand in the questionnaire, your responses can no longer be linked to you.

Results
After the study, we will analyze the results and publish the findings in a scientific article. Once published, we will share the results on our website: https://erasmusmcfoundation.nl/muziekalsmedicijn/. We expect this to happen within 12–18 months (by mid-2027 at the latest).

Questions?
If you have any questions about the study, please contact the lead researcher Michel Koopmans at:
m.r.koopmans@erasmusmc.nl

<u>Part 1: general questions</u>

Below are some general questions about your situation. Please answer the questions.

What is your age? ______

What is your gender?

- ○ Male
- ○ Female
- ○ I prefer not to say.
- ○ Other please specify: ____

Why are you currently in rehabilitation? Please give the main reason.

- ○ COPD
- ○ Asthma
- ○ Pulmonary fibrosis
- ○ Pulmonary hypertension
- ○ Lung cancer
- ○ (repeated) pneumonia
- ○ Bronchiectasis
- ○ Lung transplant
- ○ Other please specify: ____

What is the highest level of education you have completed?

- ○ Primary school
- ○ Lower vocational education or similar (such as LTS, domestic science school, lower agricultural education)
- ○ MAVO, VMBO, or similar
- ○ HAVO, VWO, or similar
- ○ Secondary vocational education or similar (such as MBO, MEAO, middle agricultural education)
- ○ Higher vocational education or similar (such as HBO, HTS, HEAO)
- ○ University
- ○ Other, please specify: _______________________________

Are you receiving inpatient or outpatient rehabilitation?

- ○ Inpatient. I stay in the rehabilitation center.
- ○ Outpatient. I stay at home, but come to the rehabilitation center for treatment.

How far are you in your rehabilitation program? Please complete the sentence below.

I am currently in week _____ of a total of _____ weeks of rehabilitation.

Do you use inhaled medication every day (such as inhalers or nebulizers)?

- ○ Yes
- ○ No

How many doses of inhaled medication do you use regularly each day? Add up the total number of doses.	

How many extra doses of inhaled medication (rescue medication) do you use per day? Add up the total number of extra doses.	

How many different types of medication do you use in total? (Not only for your lung disease, but for all conditions.)

- ○ 0
- ○ 1-2
- ○ 2-4
- ○ 5-6
- ○ 6-8
- ○ >8

Please indicate how much you agree or disagree with the statements below.

	Fully disgree	Disagree	Parly disagree	Neutral	Partly agree	Agree	Fully agree
I see myself as an enthusiast of arts and culture	○	○	○	○	○	○	○
I see myself as an enthusiast of music.	○	○	○	○	○	○	○

Are you a professional musician?	○ Yes	○ No

	Never	Several times a month	Several times a week	On a daily basis
How often do you play an instrument?	○	○	○	○
How often do you sing?	○	○	○	○

Below are several statements about your music listening style. Please indicate how much you agree or disagree with each statement.

	Fully disagree	Disagree	Agree	Fully agree
I feel when listening to music I can understand the emotions the writer performer is trying to express.	○	○	○	○
When listening to music, I have thoughts about the emotional state of the writer/composer at the time.	○	○	○	○
I like hearing the different layers of instruments and voices in a song/piece of music.	○	○	○	○
I often wonder how the mechanics of musical instruments work.	○	○	○	○

Part 2: breathlessness.

Here are some questions about your shortness of breath. First answer the questions, and then indicate how much you agree or disagree with the statements below.

Please rate your average level of dyspnea during the day.

0	0.5	1	2	3	4	5	6	7	8	9	10
Nothing at all	Very, very slightly	Very slight	Slight	Moderate	Somewhat severe	Severe		Very severe		Very, very severe.	Maximal

Please rate your average rate of perceived exertion during the day.

0	0.5	1	2	3	4	5	6	7	8	9	10
None	Very, very light	Very light	Light	Moderate	A little intense	Intense		Very intense		Very, very intense	Maximal

	Fullly disagree	Disagree	Agree	Fully agree
I have personal control over how short of breath I feel.	○	○	○	○
I know enough ways to make myself feel less short of breath.	○	○	○	○
I am open to new ways to feel less short of breath.	○	○	○	○
I am willing to do things differently if it helps with my shortness of breath.	○	○	○	○

What things, other than medication, do you use to help yourself feel less short of breath?

(write down as many as possible)

Part 3 Music and breathlessness.

Please indicate how much you agree or disagree with each statement.

	Fully disagree	Disagree	Agree	Fully agree
I think that Listening to music can help reduce my feeling of breathlessness.	○	○	○	○
I think that Singing can help reduce my feeling of breathlessness.	○	○	○	○
I think that making music can help reduce my feeling of breathlessness.	○	○	○	○
I can handle my breathlessness better when I listen to music.	○	○	○	○
I think listening to music is helpful for me when I feel breathless.	○	○	○	○
I would recommend listening to music to others who feel breathless.	○	○	○	○

	Fully disagree	Disagree	Agree	Fully agree
Listening to music distracts me from my breathlessness.	○	○	○	○
Listening to music relaxes me.	○	○	○	○
When I listen to music, I generally experience more positive feelings.	○	○	○	○
When I listen to music, I can focus better on my task.	○	○	○	○
When I listen to music, I breathe along with the rhythm.	○	○	○	○

How much benefit do you expect from listening music for the alleviation of breathlessness?

0. No benefit — 5. Neutral — 10. Maximum benefit

○ ○ ○ ○ ○ ○ ○ ○ ○ ○ ○

Imagine you feel short of breath. You want to try if listening to music helps. What kind of music would you choose? Please tick one.

- ○ Rhythmic upbeat music.
- ○ Non-rhythmic music, like meditation sounds.
- ○ My own favourite music.
- ○ Music that my healthcare providers advise me to listen to.
- ○ Other, namely: ______________________________

Listening to music may influence how well you are able to move. This can apply, for example, when doing exercise routines, walking, climbing stairs, or performing physical tasks such as self-care or household chores. The following questions are about the effect of music on movement. Please indicate how much you agree or disagree with each statement.

	Fully disagree	Disagree	Agree	Fully agree
I think that listening to music helps me to exercise better.	○	○	○	○
I think I can perform better when I listen to music during exercise.	○	○	○	○
I think I push myself too hard when I listen to music while exercising. I train too much, and that works against me.	○	○	○	○
I would recommend listening to music during exercise to people in the same situation as me.	○	○	○	○
When you listen to music while exercising I start to move with the rhythm of the music.	○	○	○	○

How much benefit do you expect from listening music during exercise?

0. no benefit — 10. Maximum benefit

○ ○ ○ ○ ○ ○ ○ ○ ○ ○ ○

Imagine: you are at the physiotherapist. You want to try if music helps you move better. What kind of music would you choose?
○ Rhythmic upbeat music.
○ Non-rhythmic music, like meditation sounds.
○ My own favourite music.
○ Music that my healthcare providers advise me to listen to.
○ Other, namely: ____

Part 4. Disadvantages of Listening to music.

Listening to music carries very few risks. However, there are reasons why some people may not always find it enjoyable. The following questions explore any potential negative effects of listening to music.

	**Fully disagree**	**Disagree**	**Agree**	**Fully agree**
Listening to music with headphones makes it harder for me to make contact with people around me.	○	○	○	○
Listening to music, when it is playing in the room, makes it harder for me to make contact with people around me.	○	○	○	○
Listening music makes me less aware of my surroundings	○	○	○	○
Music makes it harder for me to concentrate.	○	○	○	○
If I listen to music, when I feel bad, I might start to link that music to negative feelings.	○	○	○	○
I find it uncomfortable to wear earphones.	○	○	○	○
I find it uncomfortable to wear headphones.	○	○	○	○
I can get annoyed when music is playing in the room.	○	○	○	○

How much do you expect the treatment will limit your day-to-day activities (e.g. at home, at work, in the family)?										
0. Not at all										10. Extremely
○	○	○	○	○	○	○	○	○	○	○

Part 5: music and sports:

Here are some questions about how much time you spend on physical activity and music. Please answer the questions.

**How much time per week do you averagely spend on…?**	**Almost never**	**Maximum 1 hour**	**Maximum 3 hours**	**Maximum 5 hours**	**More than 5 hours**
…exercise at home, on your own?	○	○	○	○	○
…exercise outside, such as walking or cycling, without guidance from a physiotherapist or trainer?	○	○	○	○	○
…exercise in a group or with a physiotherapist/trainer?	○	○	○	○	○
**How much time per day do you averagely spend listening to music…**					
…while you are doing something else (for example, cleaning, working, driving, etc.)?	○	○	○	○	○
…while you are not doing anything else (you sit down and listen to music on purpose)?	○	○	○	○	○
**Is there music playing, or do you put on music when you…**	**Almost never**	**Less then half of the time**	**About half of the time**	**More then half of the time**	**Almost always**
…exercise at home, on your own?	○	○	○	○	○
…exercise outside, such as walking or cycling, without guidance from a physiotherapist or trainer?	○	○	○	○	○
…exercise in a group or with a physiotherapist/trainer?	○	○	○	○	○

When you listen to music, which platform do you usually use?

- ○ I do not listen to music.
- ○ I usually use a CD player or record player.
- ○ I usually listen to music on online audio streaming services (such as iTunes or Spotify).
- ○ I usually listen to music on online video streaming services (such as YouTube or TikTok).
- ○ I usually listen to music on the radio.
- ○ I usually listen to live music.
- ○ Other, please specify: ____________________________________

When you listen to music, how do you prefer to listen to it?

- ○ I never listen to music.
- ○ I prefer to play music out loud on speakers in the room.
- ○ I prefer to use earbuds.
- ○ I prefer to use headphones.
- ○ Other, please specify: ____________________________________

The questionnaire ends with some general questions. Please answer the questions.

	Yes	No
Have you ever been offered any form of music therapy as part of your treatment?	○	○
If you answered "yes" to the previous question: did you use it?	○	○
Have you ever used listening to music as a way to treat your shortness of breath?	○	○
Have you ever used singing as a way to treat your shortness of breath?	○	○
Would you be willing to take part in a treatment with music therapy if it were offered to you?	○	○
Would you be willing to take part in a study to look at the effect of music in pulmonary rehabilitation?<br>(This question is only to check your interest. If you answer "yes", you are not committing to anything. We cannot see who gives this answer and will not contact you based on your response.)	○	○
Has this questionnaire encouraged you to try using music yourself to help with your shortness of breath or to move more easily?	○	○

Do you have any other comments you would like to share with the researcher? If so, please write them here.

This is the end of the questionnaire. Thank you very much for completing it. Your contribution is greatly appreciated!

COMPOSITION-questionnaire

For health care personnel

Dear Sir/Madam,

Welcome and thank you for your participation.

Purpose of the study

This questionnaire looks at your expectations of non-medication, complementary treatments to improve shortness of breath or to help patients move more easily. We ask the same questions to both patients and you to understand differences in expectations between them.

How does it work?

You will complete a questionnaire once, which has three parts. The questionnaire starts with some general questions. After that, we ask for your opinion about non-medication, complementary treatments.

Please try to give the answer that first comes to mind. There are no right or wrong answers. Even if you have not thought about these topics before, your opinion is still valuable.

You can choose to fill in the questionnaire yourself or have someone from the research team ask you the questions. It takes 5–10 minutes to complete. Your answers will help us improve care for patients with lung disease.

Risks

There are no risks involved in this study.

Data

The questionnaire is completely anonymous, and your answers cannot be linked to you. The data will be stored safely.

Important to know

We hope that your answers will help improve care in the future. You can stop the study at any time without giving a reason. You may also skip one or more questions.

We do not collect personal data. The questionnaire is completely anonymous. Once you submit it or give it to the researcher, your answers cannot be traced back to you.

Results

After the study, we will analyze the results. We will describe the findings in a scientific article. When the results are published, we will also post this on our website:
https://erasmusmcfoundation.nl/muziekalsmedicijn/

We expect this to happen within 12–18 months (by mid-2027 at the latest).

Questions?

If you have any questions about the study, you can contact the lead researcher, Michel Koopmans:
Email: m.r.koopmans@erasmusmc.nl

Part 1: general questions

Here are some general questions about your situation. Please answer the questions.

What is your age? ______

What is your gender?

- ○ Male
- ○ Female
- ○ I prefer not to say.
- ○ Other please specify: ____

What is your current coccupation?

- ○ Pulmonologist
- ○ Rehabilitation physician
- ○ Resident doctor
- ○ Physiotherapist
- ○ Occupational therapist
- ○ Speech therapist
- ○ Exercise therapist
- ○ Creative arts therapist
- ○ Dietitian
- ○ Other, please specify: ______________________________________

Please indicate how much you agree or disagree with the statements below.

	Fully disgree	Disagree	Parly disagree	Neutral	Partly agree	Agree	Fully agree
I see myself as someone who likes art and culture.	○	○	○	○	○	○	○
I see myself as someone who likes music.	○	○	○	○	○	○	○

Are you a professional musician?	○ Yes ○ No

	Never	Several times a month	Several times a week	On a daily basis
How often do you play an instrument?	○	○	○	○
How often do you sing?	○	○	○	○

Below are several statements about your music listening style. Please indicate how much you agree or disagree with each statement.

	Fully disagree	Disagree	Agree	Fully agree
I feel when listening to music I can understand the emotions the writer performer is trying to express.	○	○	○	○
When listening to music, I have thoughts about the emotional state of the writer/composer at the time.	○	○	○	○
I like hearing the different layers of instruments and voices in a song/piece of music.	○	○	○	○
I often wonder how the mechanics of musical instruments work.	○	○	○	○

When you listen to music, which platform do you usually use?

- ○ I do not listen to music.
- ○ I usually use a CD player or record player.
- ○ I usually listen to music on online audio streaming services (such as iTunes or Spotify).
- ○ I usually listen to music on online video streaming services (such as YouTube or TikTok).
- ○ I usually listen to music on the radio.
- ○ I usually listen to live music.
- ○ Other, please specify: ______________________________________

When you listen to music, how do you prefer to listen to it?

- ○ I never listen to music.
- ○ I prefer to play music out loud on speakers in the room.
- ○ I prefer to use earbuds.
- ○ I prefer to use headphones.
- ○ Other, please specify: ______________________________________

Part 2: Expectations of music as a treatment

Please indicate how much you agree or disagree with the statements below.

	Fully disagree	Disagree	Agree	Fully agree
Listening to music can help reduce my feeling of breathlessness.	○	○	○	○
Singing can help reduce my feeling of breathlessness.	○	○	○	○
Making music can help reduce the feeling of breathlessness.	○	○	○	○
I think listening to music is helpful for my patient when heshe feels out of breath.	○	○	○	○
I would recommend listening to music to my patients when they feel out of breath.	○	○	○	○

	Fully disagree	Disagree	Agree	Fully agree
Listening to music helps to be distracted from breathlessness.	○	○	○	○
Listening to music relaxes.	○	○	○	○
When I listen to music, I generally experience more positive emotions.	○	○	○	○
When I listen to music, I can focus better on my task.	○	○	○	○
When I listen to music, I breathe along with the rhythm.	○	○	○	○

How much benefit do you expect from listening music for the alleviation of breathlessness?

0. No benefit					5. Neutral					10. Maximum benefit
○	○	○	○	○	○	○	○	○	○	○

Imagine you feel short of breath. You want to try if listening to music helps. What kind of music would you choose? Please tick one.

- ○ Rhythmic upbeat music.
- ○ Non-rhythmic music, like meditation sounds.
- ○ My own favourite music.
- ○ Music that my healthcare providers think is most suitable.
- ○ Other, namely: ____

	Fully disagree	Disagree	Agree	Fully agree
I think that listening to music helps my patient to exercise better.	○	○	○	○
I think my patient can perform better when I listen to music during exercise.	○	○	○	○
I think my patient exercise too hard when they listen to music while exercising. He/she will train too hard, and that works against him/her.	○	○	○	○
I would recommend listening to music during exercise to my patients in pulmonary rehabilitation.	○	○	○	○
When you listen to music while exercising you start to move with the rhythm of the music.	○	○	○	○

*How much benefit do you expect from listening music during exercise?

0. no benefit ○ ○ ○ ○ ○ ○ ○ ○ ○ ○ ○ 10. Maximum benefit

*Imagine: you are at the physiotherapist. You want to try if music helps you move better. What kind of music would you choose?

- ○ Rhythmic upbeat music
- ○ Non-rhythmic music, like meditation sounds
- ○ My own favourite music
- ○ Music that my healthcare providers think is most suitable
- ○ Other, namely: ____

	Fully disagree	Disagree	Agree	Fully agree
Listening to music with headphones makes it harder for my patients to connect with people around them.	○	○	○	○
Listening to music, when it is playing in the room, makes it harder for my patients to connect with people around them.	○	○	○	○
Listening music makes the patient less aware of my surroundings	○	○	○	○
Music makes it harder for my patients to concentrate.	○	○	○	○
When my patient listens to music, when he/she feel bad, he might start to link that music to negative feelings.	○	○	○	○
It is uncomfortable to wear earphones.	○	○	○	○
It is uncomfortable to wear headphones.	○	○	○	○
I get annoyed when music is playing in the room.	○	○	○	○
**I think that using music as a treatment can add value for patients with lung disease.**	○	○	○	○

How much do you expect a treatment with music will limit the day-to-day responsibiliteits of our patients (e.g. at home, at work, in the family)?

0. Not at all ○ ○ ○ ○ ○ ○ ○ ○ ○ ○ ○ 10. Extremely

Has this questionnaire encouraged you to try using music during your treatment?

- ○ Yes
- ○ No

This is the end of the questionnaire. Thank you for completing it!
